# Cardiovascular Disease, Mitochondria, and Traditional Chinese Medicine

**DOI:** 10.1155/2015/143145

**Published:** 2015-05-17

**Authors:** Jie Wang, Fei Lin, Li-li Guo, Xing-jiang Xiong, Xun Fan

**Affiliations:** ^1^Department of Cardiology, Guang'anmen Hospital, China Academy of Chinese Medical Sciences, Beijing 100053, China; ^2^Clinical Medical College, Hubei University of Chinese Medicine, Wuhan 430065, China

## Abstract

Recent studies demonstrated that mitochondria play an important role in the cardiovascular system and mutations of mitochondrial DNA affect coronary artery disease, resulting in hypertension, atherosclerosis, and cardiomyopathy. Traditional Chinese medicine (TCM) has been used for thousands of years to treat cardiovascular disease, but it is not yet clear how TCM affects mitochondrial function. By reviewing the interactions between the cardiovascular system, mitochondrial DNA, and TCM, we show that cardiovascular disease is negatively affected by mutations in mitochondrial DNA and that TCM can be used to treat cardiovascular disease by regulating the structure and function of mitochondria via increases in mitochondrial electron transport and oxidative phosphorylation, modulation of mitochondrial-mediated apoptosis, and decreases in mitochondrial ROS. However further research is still required to identify the mechanism by which TCM affects CVD and modifies mitochondrial DNA.

## 1. Introduction 

At present, the anatomical paradigm of medicine and the Mendelian paradigm of genetics have failed to interpret anticipated genetic causes of common age-related diseases that include diabetes and metabolic syndrome, Alzheimer's disease, Parkinson's disease, cardiovascular disease (CVD), and cancer [1]. Nevertheless, with the development of medicine, mitochondrial biology and genetics have become excellent candidates for expanding these anatomical and Mendelian paradigms to reveal the complexities of CVDs that have become a worldwide problem [2]. Thus, the mitochondrial paradigm (a complementary concept to Mendelian genetics) is a paradigm of CVD susceptibility and cellular function [3].

Mitochondria are linked to the cardiovascular system. The heart is highly dependent for its function on oxidative energy generated in mitochondria, primarily by fatty acid beta-oxidation, the respiratory electron chain, and oxidative phosphorylation. The ability to utilize oxygen drives the development and evolution of the cardiovascular system in multicellular organisms [4]. Mitochondria are evolutionary endosymbionts derived from bacteria and contain DNA similar to bacterial DNA. Restructuring of the protomitochondrial genome included the transfer of virtually all 1500 genes of the mitochondrial genome into chromosomal nuclear DNA; mitochondrial DNA (mtDNA) retains 13 polypeptide-encoding genes, 2 rRNA genes, and 22 tRNA genes [5]. For those mtDNA-encoded proteins is either an electron or a proton carrier of oxidative phosphorylation. Mitochondria are recognizing sensors of oxygen and fuel and producers of heat and ATP. They generate reactive oxygen species, acting as signaling hubs with their redox-based signals reaching the cell membrane and the nucleus, and they regulate calcium and effective inducers of cell death (apoptosis) [5]. Notably, mtDNA deletion is significantly associated with loss of atrial adenine nucleotides. Atrial concentrations of ATP, ADP, AMP, and total adenine nucleotides were significantly lower in patients with deletions than those in patients without deletions [6].

Mutations of mtDNA are associated with several clinical manifestations affecting different systems. By virtue of the functional role of mitochondria in energy metabolism and reactive oxygen species production, mutations in mtDNA are potential candidate risk factors for cardiovascular disorders. This has led to the mitochondrial paradigm in which it has been proposed that mtDNA sequence variation contributes to susceptibility to CVD. In addition, defects in mitochondrial structure and function are associated with CVDs, such as dilated and hypertrophy cardiomyopathy, cardiac conduction defects and sudden death, ischemic and alcoholic cardiomyopathy, and myocarditis [7].

Traditional Chinese medicine (TCM) has been in use for over 2500 years and has historically established itself as a system of holistic medical care in China [8]. What is more, Chinese medicine and integrative medicine health provision in conventional medical clinics and hospital settings have emerged worldwide [9–11]. TCM is known to efficiently prevent and cure CVD and other illnesses, as well as have a habilitative, strengthening effect on the body. However, the mechanisms by which TCM alleviates CVD are not clearly understood. Now unrecognized mitochondrial pathways and new therapeutic strategies for the treatment of CVD by TCM are systematic survey. If this strategy proves successful, it may have been prescient that a major concept in the parlance of Mitochondria is chi, which loosely translates as vital force or energy [5].

## 2. Coronary Artery Disease 

Coronary artery disease (CAD) is one of the most widespread and common causes of death in the world. It is a multifactorial process that appears to be caused by the interaction of environmental risk factors with multiple predisposing genes. An increase in oxidative stress in CVD may be responsible for the accumulation of mtDNA damage in CAD patients. Reactive oxygen species (ROS) can damage mtDNA and this may cause tissue dysfunctionality, leading to early events in CVD.

Recent evidence suggests that a specific mtDNA deletion of 4977 bp, 3243A>G, and 16189T>C is associated with myocardial dysfunction and bioenergetic deficits. In numerous studies, a significant higher incidence of mtDNA 4977 was observed in CAD patients than in healthy subjects, and the relative degree of deletion was higher in CAD patients than in the control group [12]. Relative quantifications showed that the amount of mtDNA (4977) deletion was greater and that telomere length was shorter in CAD patients [13]. Of the most conventional risk factors, smoking and dyslipidemia have the strongest association with the degree of mtDNA (4977) deletion and significantly correlate with telomere attrition [13]. In addition, cardiac diseases are frequently detected among sudden natural deaths, with mtDNA 4977-deletion present more frequently in victims of sudden natural death than in subjects who died of unnatural causes [14].

In other studies, mitochondrial dysfunction may affect autonomic regulatory systems more directly; the A3243G mitochondrial DNA mutation [15] and individuals with the 3243A>G mutation in mtDNA have abnormalities in the spectral and fractal characteristics of heart rate variability, which suggest altered cardiac autonomic regulation [16]. The mitochondrial DNA variant 16189T>C is also associated with CAD and myocardial infarction in Saudi Arabs. The impact of mtDNA polymorphism on CAD manifestation is influenced by important confounders, particularly the presence of myocardial infarction, hypertension, and age [17].

## 3. Hypertension 

Essential hypertension (EH), a polygenic, multifactorial, and highly heterogeneous disorder of unknown etiology, is the most common CVD in the world. Several studies have noted that mtDNA variation has become an additional target in the investigation of potential EH heritability. To assess the contribution of the mitochondrial genome to EH, researchers performed a systematic, extended screening of hypertensive individuals to identify potentially pathogenic mtDNA mutations. Of these mtDNA mutations, mt-transfer RNA (tRNA) was a mutational hotspot for pathogenic mutations associated with EH. Mutant mtDNA aggravates mitochondrial dysfunction, critically contributing to clinical phenotypes [18]. Moreover, the sequence of the entire mitochondrial genome in probands from 20 pedigrees was recently analyzed. Comparison with the reference “Cambridge” sequence revealed a total of 297 base changes, including 24 in ribosomal RNA (rRNA) genes, 15 in transfer RNA (tRNA) genes, and 46 amino acid substitutions [19]. The presence of the m.14484T>C mutation was reported in a Chinese family with maternally inherited EH. Mitochondrial respiration rate and membrane potential were reduced in lymphoblastoid cell lines established from affected members carrying m.14484T>C. There was a compensatory increase in mitochondrial mass in these mutant cell lines [20]. In addition, the 4435A>G mutation may act as an inherited risk factor for the development of hypertension in this Chinese pedigree. A failure in mitochondrial tRNA metabolism, caused by the 4435A>G mutation, led to an approximately 30% reduction in the rate of mitochondrial translation [21]. Furthermore, uncommon/rare variants were identified by sequencing the entire mitochondrial genome of 32 unrelated individuals with extreme hypertension and genotyping 40 mitochondrial single nucleotide polymorphisms in 7219 individuals. The nonsynonymous mitochondrial single nucleotide polymorphism 5913G>A in the cytochrome c oxidase subunit 1 of respiratory complex IV was significantly associated with blood pressure and fasting blood glucose levels [22]. In addition, the data provide support for a maternal effect on hypertension status and quantitative systolic hypertension, which is consistent with a mitochondrial influence. The estimated fraction of hypertensive pedigrees that were potentially the result of mitochondrial effects was 35.2%. Mitochondrial heritabilities for multivariable-adjusted long-term average systolic hypertension and diastolic hypertension were 5% and 4%, respectively [23].

## 4. Cardiomyopathy 

Dilated cardiomyopathy (DCM) is one of the most frequent forms of primary myocardial disease and the third most common cause of heart failure. Recent studies suggest that mtDNA mutations and mitochondrial abnormalities may be contributing factors for the development of DCM. Defects in mtDNA, both deletions and tRNA point mutations, are associated with cardiomyopathies [24]. In a study some patients had heteroplasmic mtDNA mutations [24]. Research also suggests that TNF-alpha-induced heart failure may be associated with reduced mtDNA repair activity [25]. A novel duplication in the mitochondrially encoded tRNA proline gene was found in a patient with dilated cardiomyopathy. Part of this duplication is localized within the tRNA proline gene that can act against oxidative stress and regulate the balance of reactive oxygen species within cells. The patient was described as having DCM and a novel mtDNA duplication. Sequencing of the mtDNA control region was performed, and a 15 bp duplication was observed between nucleotides 16,018 and 16,032 [26]. Five patients with CM shared a novel homoplasmic point mutation, and all of them demonstrated the evolutionarily related D-loop sequence of mitochondria [27]. At present, 13 types of mutations in subunits of the mitochondrial respiratory chain complexes are associated with cardiomyopathy and they include Cyt b 14927A>G, Cyt b 15236A>G, Cyt b 15452A>G, Co I 6521C>G, Co II 7673A>G, ND I 3394T>C, ND 6 13258A>T, and ND 6 14180T>C [28].

Additionally, left ventricular noncompaction cardiomyopathy (LVNC) is a rare congenital cardiomyopathy that is associated with mutations in mtDNA, as mtDNA copy number and mtDNA content were lower in the myocardium of LVNC patients, with abnormal mitochondrial morphology, suggesting that mitochondrial dysfunction may be associated with the etiology of LVNC [29]. In addition, mtDNA mutations in patients with beta myosin heavy chain- (beta MHC-) linked hypertrophic cardiomyopathy (HCM) are present in individuals who develop congestive heart failure. Although beta MHC gene mutations may be determinants of HCM and both of the mtDNA mutations in these patients are known prerequisites for pathogenicity. Coexistence of other genetic abnormalities in beta MHC-linked HCM, including mtDNA mutations, may contribute to variable phenotypic expression and explain the heterogeneous behavior of HCM [30]. Therefore, mtDNA is a key player in the pathogenesis of cardiomyopathy and has provided new mechanism-based approaches to therapy [31].

## 5. Heart Failure 

Heart failure (HF) is the end stage of various types of CVDs whose mortality rate is considerably high. The advent of the mitochondrial paradigm has provided important insights into the mechanisms underlying HF. Results of studies show an intimate link between ROS, TNF-alpha, mtDNA damage, and defects in electron transport function, which may lead to the additional generation of ROS and might also play an important role in the development and progression of left ventricle remodeling and HF [32].

Excessive ROS produced by electron leaks from mitochondria in failing myocardium play an important role in the development and progression of HF and cardiac remodeling [33]. Mitochondrial electron transport is an enzymatic source of oxygen radical generation and is also a target of oxidant-induced damage [34]. Chronic increases in oxygen radical production in the mitochondria can possibly lead to a catastrophic cycle of mtDNA damage as well as functional decline, further oxygen radical generation, and cellular injury. ROS directly impair contractile functions by modifying proteins central to excitation-contraction coupling and activate a broad variety of hypertrophy signaling kinases and transcription factors and mediate apoptosis. Moreover, ROS stimulate cardiac fibroblast proliferation and activate matrix metalloproteinases, leading to extracellular matrix remodeling. ROS also play an important role in the pathophysiology of cardiac remodeling and heart failure [34–36]. Another study using Southern blot analysis showed that mtDNA copy number relative to a nuclear gene (18S rRNA) preferentially decreases by 44% after myocardial infarction, which was associated with a parallel decrease in the mtDNA-encoded gene transcripts, including subunits of complex I (ND1, 2, 3, 4, 4L, and 5), complex III (cytochrome b), complex IV (cytochrome c oxidase), and rRNA (12S and 16S) [32].

Therefore, oxidative stress and mtDNA damage are excellent therapeutic targets. Overexpression of peroxiredoxin-3 (Prx-3), mitochondrial antioxidants, or mitochondrial transcription factor A (TFAM) could ameliorate the decline in the mtDNA copy number in failing hearts. Consistent with alterations in mtDNA, the decrease in oxidative capacity may also be prevented [36].

ROS can damage mtDNA and thus lead to mitochondrial dysfunction and additional generation of ROS. Overexpression of TFAM, which is essential for mtDNA transcription and replication, ameliorates cardiac remodeling and failure [33]. Overexpression of TFAM attenuates the decrease in mtDNA copy number after myocardial infarction, ameliorates pathological hypertrophy, and markedly improves the chances of survival. TFAM also protects the heart from mtDNA deficiencies and attenuates left ventricular remodeling and failure after myocardial infarction created by ligating the left coronary artery [37]. Recombinant human TFAM protein increases mtDNA and abolishes the activation of nuclear factor of activated T cells (NFAT), which is well known to attenuate pathological hypertrophy of cardiac myocytes [38]. Furthermore, there are the intimate links between TNF-alpha, ROS, and mtDNA damage that might play an important role in myocardial remodeling and failure [39].

## 6. Atherosclerosis 

Atherosclerotic plaques, which contain vascular and inflammatory cells, lipids, cholesterol crystals, and cellular debris, restrict lumen size and often rupture, causing infarctions [4]. Atherosclerosis is the major risk factor for development of CVD based on arterial endothelial dysfunction and is caused by the impairment of endothelial-dependent dilation. Recent findings have shown that the level of heteroplasmy of some somatic mtDNA is associated with coronary atherosclerosis and impaired mitochondrial function. Structural and qualitative changes in mitochondrial components such as mtDNA may be directly involved in the development of multiple atherogenic mechanisms, including advanced oxidative stress, abnormalities in glucose and fat metabolism, and altered energy homeostasis [40]. Atherosclerotic vascular disease is typically a disease of aging. In accordance with the ROS theory of aging [41], accumulated data point to a key role of ROS in the pathogenesis of atherosclerosis. mtDNA, owing to electron transport chain proximity and the relative lack of mtDNA repair mechanisms, is the most vulnerable target of mitochondrial ROS. Greater mtDNA damage is present in human aorta atherosclerosis samples than in those of age-matched transplant donors [42]. Mitochondria have been recognised as critical regulators of cell death, generation of ATP, and the generation of reactive oxygen species (ROS), and mtDNA damage leads to mitochondrial dysfunction and promotes atherosclerosis directly [43]. Damage of mtDNA in the vessel wall and circulating cells is widespread and causative, indicates a higher risk of atherosclerosis, promotes atherosclerosis independently of ROS through effects on smooth muscle cells and monocytes, and correlates with higher-risk plaques in humans [44].

## 7. Summary 

At present, modes for diagnosis and treatment of CVD differentiation used by modern medicine combined with syndrome differentiation from TCM have become a main method of treatment of CVD in China and other nations [45–47]. For example,* Radix Salviae miltiorrhizae*,* Radix et Rhizoma Notoginseng*,* Rhizoma Chuanxiong*,* Radix Astragali*, and others have been used for the treatment of CVDs [48], showing a remarkable curative effect however, mechanisms remain unknown.

Human mtDNA mutations cause a large spectrum of clinically important cardiovascular events. Research suggests that if mitochondrial ROS production becomes excessive, it is possible for mitochondria and mtDNA to be damaged [5]. To detect mitochondrially active compounds, Wallace assembled a mitochondrial cDNA expression array, the MITOCHIP, which interrogates ~1000 genes involved in mitochondrial energy production, ROS biology, and apoptosis. TCM might target mitochondrial function with a serial action aimed at treating CVDs. For example, restoratives are all medicinal herbs for replenishing qi and blood, nourishing yin and yang, improving the functions of the internal organs and body immunity, and relieving the various symptoms of weakness. Historically Chinese have been taking Astragali Radix as a natural invigorant in nourishing life. Astragali Radix injection can reverse mitochondrial dysfunction and abnormal structure in myocardial cells during myocardial cell hypertrophy, caused by angiotensin II. Reversion of myocardial cell hypertrophy and the restructuring of myocardial cells help improve energy metabolism in myocardial cells [49].

A yang-invigorating Chinese herb formula treatment increased red blood cell Cu-Zn-SOD (superoxide dismutase) activity and mitochondrial ATP generation capacity and reduced glutathione and alpha-tocopherol levels. It has been suggested that yang-invigorating herbs might promote ATP generation by increasing mitochondrial electron transport and induce increases in mitochondrial antioxidant capacity in various tissues as evidenced by a reduction in the extent of ROS generation in vitro. Red cell Cu-Zn-SOD activities correlated positively with mitochondrial antioxidant component tissue levels/activity. By contrast, yin-nourishing herbs either did not stimulate or decrease myocardial ATP generation capacity [50, 51].* Herba Cistanche* belongs to the A class of yang-invigorating herbs and increases mitochondrial glutathione activity.

It increases mitochondrial ATP content, decreases mitochondrial Ca^2+^ content, and increases mitochondrial membrane potential [52].* Ganoderma lucidum* increases the activity of cardiac mitochondrial enzymes and respiratory chain complexes in aged rats [53].

Moreover, Sini Decoction (SND) increases the activity and mRNA expression of Mn-SOD and the activity of Na^+^-K^+^ ATPase and Ca^2+^-ATPase, while the degree of mitochondrial swelling and the content of malondialdehyde (MDA) were reduced in SND-treated rats [53]. Sodium tanshinone IIA sulfonate (STS) stimulates mitochondrial NADH oxidation dose dependently and partly restores NADH oxidation in the presence of a respiratory inhibitor (rotenone, antimycin A, or potassium cyanide) [54].

DaBu-Yin-Wan and QianZheng-San ameliorate behavior induced by administration of 1-methyl-4-phenyl-1,2,3,6-tetrahydropyridine (MPTP) and synergistically prevent decreases in tyrosine hydroxylase (TH) expression but also increase monoaminergic content and activity, improve ultrastructural changes, decrease mtDNA damage, and synergistically upregulate the expression of ND1 mRNA [55].

In addition, Guan-Xin-Er-Hao (GXEH) attenuates postischemia myocardial apoptosis. The antiapoptotic mechanisms of GXEH may involve mitochondrial cytochrome c-mediated caspase-3 activation in cardiomyocytes after the occurrence of acute myocardial infarction. GXEH adjusts the balance of Bax and Bcl-2 toward an antiapoptotic state, decreases mitochondrial cytochrome c release, reduces caspase-9 activation, and attenuates subsequent caspase-3 activation and postischemic myocardial apoptosis in rats [56]. Furthermore, Ginkgo biloba leaf extract alters mitochondrial gene expression, possibly by modulating mitochondrial-associated apoptosis [57]. Danshen-Gegen (DG) decoction treatment activates both ERK/Nrf2- and PKC epsilon-mediated pathways, presumably through ROS arising from CYP-catalyzed processes, with resultant inhibition of hypoxia/reoxygenation-induced apoptosis immediately after DG treatment, or even after an extended time interval following DG treatment [58]. In addition, the derivative deoxysppanone B was found to act through microtubules to increase oxidative phosphorylation and decrease mitochondrial ROS [59].

The mitochondrial paradigm for CVD susceptibility and cellular function may become a complementary concept to Mendelian genetics. In this regard, Wallace suggests that mitochondria are Qi (Chi), which loosely translates as vital force or energy, according to its TCM interpretation. The fact that TCM uses a variety of mitochondrial functional readouts may reveal previously unrecognized mitochondrial pathways and new therapeutic strategies to manipulate them, and these could then be applied to treating CVD. It is therefore important to consider how we might initiate a search for a TCM method to regulate the function of mitochondria and the effects of mtDNA to treat CVD.
